# Sustainability determinants of an intervention to identify clinical deterioration and improve childhood cancer survival in Latin American hospitals: the INSPIRE study protocol

**DOI:** 10.1186/s43058-023-00519-y

**Published:** 2023-11-17

**Authors:** Virginia McKay, Bobbi Carothers, Dylan Graetz, Sara Malone, Maria Puerto-Torres, Kim Prewitt, Adolfo Cardenas, Yichen Chen, Meenakshi Devidas, Douglas A. Luke, Asya Agulnik

**Affiliations:** 1 Brown School, Washington University in St. Louis, St. Louis, MO USA; 2 Department of Global Pediatric Medicine, St. Jude Children’s Research Hospital, Memphis, TN USA; 3 Division of Population Health Science, Washington University in St. Louis School of Medicine, St Louis, MO United States; 4 Division of Critical Care, St. Jude Children’s Research Hospital, Memphis, TN USA

**Keywords:** Pediatric cancer, Pediatric Early Warning Systems (PEWS), Implementation science, Sustainability, Low- and middle-income countries, Resource-poor settings

## Abstract

**Background:**

More than 90% of children with cancer live in low-resourced settings, where survival is only 20%. Sustainable evidence-based (EB) interventions yielding ongoing beneficial patient outcomes are critical to improve childhood cancer survival. A better understanding of factors promoting intervention sustainability in these settings is urgently needed. The aim of this study is to provide an empirical understanding of how clinical capacity for sustainability, or the resources needed to sustain an intervention, impacts the sustainment of Pediatric Early Warning System (PEWS), an EB intervention that improves pediatric oncology outcomes in low-resource hospitals by detecting clinical deterioration and preventing the need for more intense treatment.

**Methods:**

We will conduct a prospective, longitudinal study of approximately 100 resource-variable hospitals implementing and sustaining PEWS participating in Proyecto EVAT, a quality improvement collaborative of Latin American pediatric oncology centers. Aim 1: We will evaluate how clinical capacity for sustainability changes over time through 5 to 9 prospective measurements of capacity via survey of clinical staff using PEWS (approximately *n* = 13 per center) during the phases of PEWS adoption, implementation, and sustainability using the Clinical Sustainability Assessment Tool (CSAT). Aim 2: We will determine the relationship between capacity and a) PEWS sustainment and b) clinical deterioration mortality among pediatric oncology patients at centers sustaining PEWS for 2 to 10 years using chart review and an existing patient outcomes registry. Aim 3: We will develop novel strategies to promote sustainability by gaining a deeper understanding of perceived challenges to building capacity and PEWS sustainment. In combination with quantitative outcomes, we will conduct 24 focus groups with staff (doctors, nurses, and administrators) from hospitals with both high (*n* = 4) and low capacity (*n* = 4). We will then use implementation mapping to generate theoretically driven, empirically-supported sustainability strategies.

**Discussion:**

This study will advance implementation science by providing a theoretically driven, foundational understanding of factors that predict sustainability among a large, diverse cohort of hospitals. We will then use this knowledge to develop sustainability evidence-informed strategies that optimize capacity and promote long-term sustainment of PEWS and improvements in patient outcomes, thus promoting equity in childhood cancer care globally.

Contributions to the literature

- This study evaluates the ability of resource-variable hospitals in Latin America to sustain an intervention that improves outcomes among hospitalized children with cancer thus improving survival for children with cancer globally.
- A better understanding of factors contributing to the sustainability of evidence-based interventions is urgently needed. This study will identify components of clinical capacity that contribute to the long-term sustainability of an evidence-based intervention in resource-variable hospitals.
- This study will develop one of the few evidence-informed strategies to promote sustainability, thus advancing implementation science.

## Background

While much of implementation science focuses on adopting and implementing evidence-based interventions, sustainability is the least studied phase of the implementation continuum [1, 2]. Ideally, interventions should be sustained unless they are no longer effective or more effective interventions become available [3–5]. Many interventions are abandoned when they should be continued, often when external support, such as grant funding or collaborative assistance, is removed [6–9]. Implementing interventions is costly, and if interventions are prematurely abandoned, then initial investments are lost [10, 11]. This is especially problematic in low-resource settings where there are few opportunities to implement new interventions. Most importantly, evidence-based interventions that are not sustained cannot provide continued health benefits to patients.

The current body of scientific literature focuses primarily on conceptualizing and theorizing sustainability in health [11, 12]. A general consensus within this literature establishes the relationship between the immediate context where interventions are implemented and the likelihood of intervention sustainability [12]. Clinical capacity for sustainability, which characterizes the immediate context, is defined as the resources necessary to sustain an intervention and includes engaged staff, leadership and stakeholders, organizational readiness, workflow integration, implementation and training, and monitoring and evaluation as the most proximal contextual determinants influencing intervention sustainment [10, 13, 14]. While there are several conceptual frameworks identifying sustainability determinants, few have been evaluated empirically. A recent review of determinants of hospital intervention sustainability included no studies from low-income countries, and two-thirds of the studies were qualitative [15]. Another notable gap is a lack of theoretically informed, empirically driven sustainability strategies to modify determinants and promote intervention sustainability. A recent review of 62 sustainability strategies for health interventions noted the majority were strictly conceptual frameworks and only two were active strategies to either plan for sustainability or promote sustainability after implementation in acute care settings [11]. This existing work highlights a lack of comprehensive evaluation of factors and strategies that promote sustainability in low-resource settings, a meaningful knowledge gap that will be addressed in the current study.

This work will be conducted in the context of global pediatric oncology. The global burden of pediatric cancer is disproportionately shifted to low- and middle-income countries, which bear over 90% of childhood cancer cases [16], with a dismal survival rate of approximately 20% [17]. To reduce these disparities, the World Health Organization Global Initiative for Childhood Cancer [18] and other initiatives [19] emphasize the need to improve access to and outcomes of childhood cancer treatment globally. However, hospitals in low-resource settings frequently lack adequate infrastructure and staffing to deliver needed supportive care during cancer treatment [20–22], resulting in late identification of clinical deterioration events (CDEs) and high rates of preventable deaths [23, 24]. Our prior work in Latin America demonstrated high rates of CDEs among hospitalized children with cancer and a 30% mortality rate in patients with deterioration [25]. This illustrates an urgent need for effective, low-cost, and sustainable supportive care interventions, including strategies for timely identification of CDEs, to improve global childhood cancer survival. Two key challenges persist in addressing this imperative: (1) successful implementation of evidence-based interventions and (2) long-term sustainability of implemented interventions. The latter is the focus of this proposed study.

### Pediatric Early Warning Systems (PEWS)

To more rapidly identify CDEs, many hospitals use PEWS: nursing-administered bedside clinical acuity scoring tools associated with escalation algorithms. PEWS accurately predict the need for pediatric intensive care unit (PICU) transfer in pediatric oncology patients in high-resource hospitals [26, 27]. Escala de Valoración de Alerta Temprana (EVAT) is a Spanish-language PEWS adapted for low-resource settings. EVAT includes a 5-component scoring tool (neurologic, cardiovascular, respiratory, staff and family concern) based on vital signs, physical examination findings, and treatment requirements [28]. Hospitalized patients are scored 0 to 11 using the PEWS tool by a bedside nurse during routine vital sign assessments. Higher scores indicate potential clinical deterioration and are addressed following an action algorithm that guides the clinical team in appropriate escalation of care. In 2014, Dr. Agulnik worked with local stakeholders to implement and validate this PEWS at a low-resource pediatric oncology hospital in Guatemala [28–30], resulting in a 27% reduction in CDEs, optimized PICU utilization [30], improved interdisciplinary communication, provider empowerment and perceived quality of care [31–33], and an annual cost-savings of over US$350,000 [34].

### Proyecto EVAT implementation strategy

These results led St. Jude Global [19] at St. Jude Children’s Research Hospital to establish Proyecto EVAT, a quality improvement collaborative to improve survival in hospitalized children with cancer in Latin America [19, 35]. Hospitals that care for children with cancer are recruited to Proyecto EVAT through collaboration with the St. Jude Global Alliance [19] or via learning about the program from others. Hospitals apply to an annual cohort, obtain institutional approval to participate, and are assigned to one of twelve mentor training centers. Each hospital assembles a local PEWS implementation leadership team, including at minimum a pediatric oncology nurse, pediatric oncology ward physician, and intensivist, adjusting the size to local needs.

Proyecto EVAT hospitals are guided through a 3-phase implementation process via bimonthly virtual mentorship meetings. During the planning phase, hospitals implement a de-identified prospective registry of CDEs in pediatric oncology patients, collecting 6–12 months of baseline data. To maintain effectiveness, fidelity with no changes is recommended to the validated components of the PEWS tool [28, 36]. Validated components of PEWS considered to be essential to its effectiveness include the scoring system, an algorithm, and using the tool with every vital sign assessment. Hospitals, however, are encouraged to adapt other elements of PEWS to their setting, including adjusting the wording of the PEWS tool and details of the PEWS algorithm to better fit with local medical language, available resources, and processes for care escalation in hospitalized children [37].

After completing these activities, hospitals move to the implementation phase. Experts from St. Jude and the mentor centers teach local implementation teams PEWS implementation strategies using a standardized curriculum. Implementation teams then conduct local training with clinicians, pilot PEWS, and assess its effectiveness. From the start of the pilot, local leaders track PEWS use and fidelity (measured by the three types of PEWS errors) and patient outcomes (CDE registry), which are sent to St. Jude monthly. Implementation is considered successful (i.e., implementation completion) when a hospital achieves sufficient PEWS use and fidelity, defined as < 15% PEWS errors for two consecutive months. Hospitals then move to the sustainability phase, with the expectation of indefinite PEWS sustainment through continued PEWS use and fidelity, resulting an ongoing positive impact on patient outcomes. During this phase, hospitals continue collaborating with Proyecto EVAT through monthly virtual meetings and/or as mentor centers.

Since 2017, regional enthusiasm for Proyecto EVAT has grown, with 10–15 new hospitals enrolling in the program annually and more expressing interest. In an ongoing evaluation of PEWS at participating hospitals, we found that local implementation leadership teams successfully overcame implementation barriers and initially achieved excellent PEWS fidelity as well as improvements in patient outcomes, including a reduction in deterioration morality in participating centers [38–44].

### Challenges sustaining PEWS

Preliminary data from Proyecto EVAT demonstrates hospitals improve clinical capacity during implementation. Hospitals may struggle to sustain PEWS, but adequate clinical capacity supports PEWS sustainment. In a preliminary analysis of hospitals using PEWS for up to 24 months, approximately 30% reported PEWS error rates above the 15% threshold for one or more months, indicating a lack of PEWS sustainment. A qualitative study of barriers and enablers to PEWS implementation at five Proyecto EVAT hospitals sustaining PEWS demonstrated several capacity-related barriers to sustainability, including the COVID-19 pandemic, fluctuations in human and material resources needed for PEWS, staff turnover resulting in insufficient training, difficulty obtaining leadership buy-in, and lack of internal systems for ongoing PEWS monitoring [45–47]. After controlling for individual, hospital, and intervention factors, clinical capacity as measured by the Clinical Sustainability Assessment Tool (CSAT) was significantly associated with PEWS sustainment (OR 3.27, *p* < 0.0001). Marginal effects from the final model indicate that an increasing capacity score was positively associated with PEWS sustainment (11% greater likelihood of sustainment for every additional CSAT point on a scale from one to five). These results suggest that not all hospitals have sufficient capacity for sustainability, a notable portion do not sustain PEWS, and higher clinical capacity makes PEWS sustainment more likely. However, the relationship between capacity and sustainment may be dynamic over time and longitudinal examinations are needed to understand these relationships. The *IN*vestigating *S*ustainability of *P*EWS In *RE*source-limited hospitals (INSPIRE) study will build on this prior work by examining clinical capacity beyond implementation to understand its impact on PEWS sustainability over time.

## Methods and design

The INSPIRE study attempts to answer the overall question of what are relevant components of clinical capacity that determine PEWS sustainability in resource-variable hospitals providing childhood cancer care (Table 1). To answer this question, we will conduct a longitudinal observational study of a cohort of pediatric oncology centers implementing and sustaining PEWS over five years. Upon completing this study, we will establish how clinical capacity changes over time (Aim 1), determine the influence of capacity on sustainment and patient outcomes (Aim 2), and use a mixed-method approach to understand staff perspectives on challenges to capacity building and sustainability and develop novel strategies to promote sustainability (Aim 3).

**Table 1:** Overview of study aims

**Overall research question**: What are the relevant components of capacity that determine PEWS sustainability in low-resource hospitals?		
**Aim 1:** Changes in capacity over time	Question	How does capacity for sustainability and its components change over time through the phases of PEWS adoption, implementation, and sustainability?
	Hypothesis	Capacity develops during early implementation and increases over time
**Aim 2:** Sustainment and patient outcomes	Questions	How do changes in overall capacity and its components predict (a) PEWS sustainment or abandonment and (b) improvement in patient outcomes?
	Hypothesis	Capacity and its components predict PEWS sustainment and continued reduction in clinical deterioration event mortality over time
**Aim 3:** Develop sustainability strategies	Questions	What are staff perceptions of challenges to capacity development and their impact on PEWS sustainability in low-resource hospitals? What are potential strategies that promote sustainability in these settings?

### EVAT steering committee and external advisory board

This study will leverage the Proyecto EVAT Steering Committee (EVAT SC), a 33-member multidisciplinary team of nurses and physicians from 12 hospitals in 9 Latin American countries. EVAT SC members are experts in PEWS implementation and are selected from regional PEWS training centers. The EVAT SC reviewed the CSAT, the primary measure for the studey, for conceptual appropriateness, was involved in the translation and piloting of the CSAT, and approved this proposed study as feasible, important, and regionally acceptable. For the duration of the proposed work, the EVAT SC will be updated on project progress twice annually to provide oversight and feedback and to ensure regional appropriateness and applicability. This work will also be supported by an External Advisory Board composed of three senior investigators with expertise in implementation research, quality improvement, and global pediatric oncology.

### Capacity and sustainability framework

**Fig. 1:**
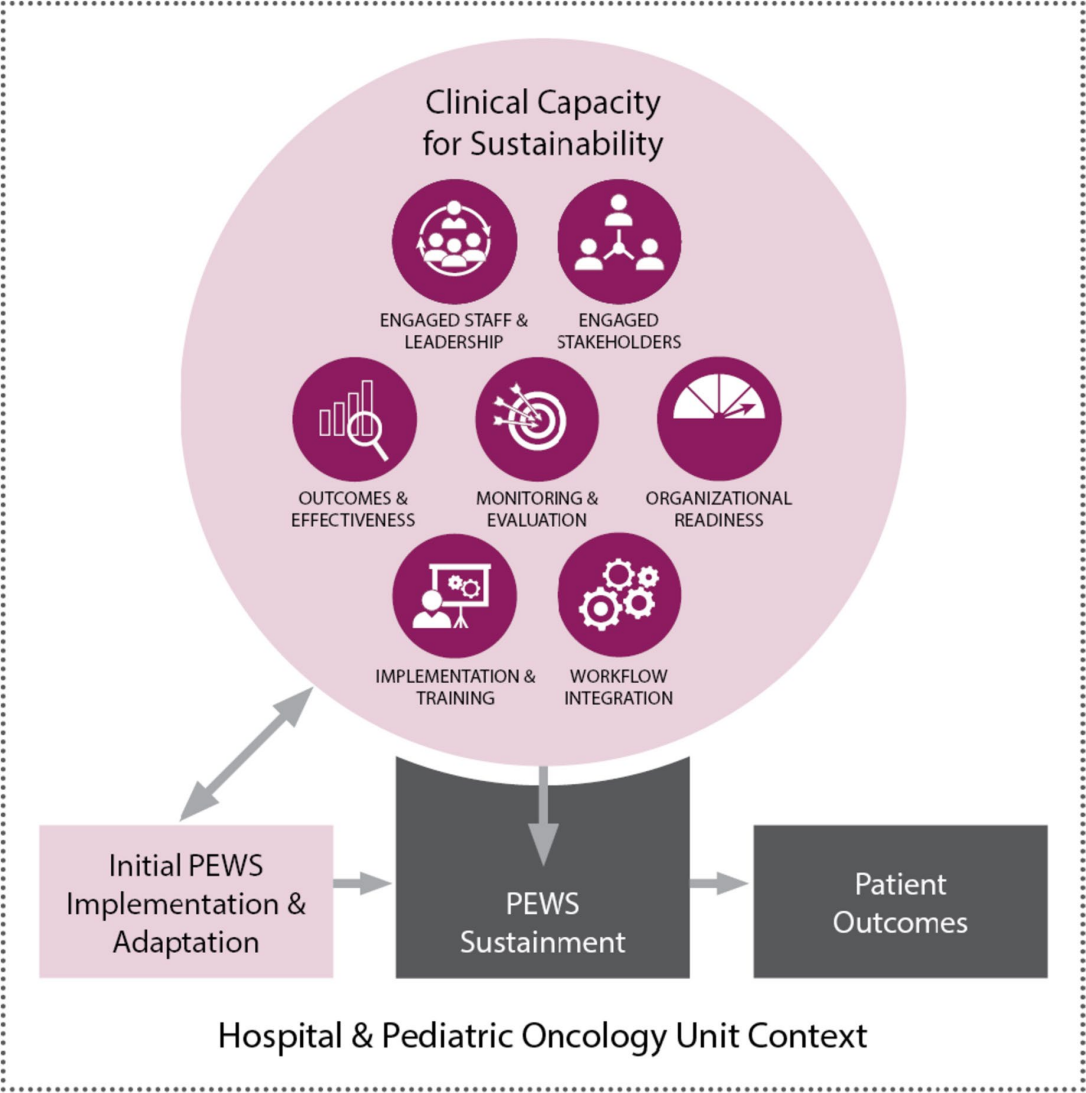
Capacity and sustainability conceptual framework

Our conceptual model (Fig. 1) is guided by the dynamic sustainability framework [14] and the public health capacity for sustainability framework [13]. The dynamic sustainability framework posits that interventions are implemented within a clinical context nested in a broad ecological system with a complex interplay between sustainability determinants, intervention sustainment, and intervention outcomes. We define *sustainment* as the continued use of evidence-based intervention elements after implementation, without external support; and *sustainability* to more broadly include intervention sustainment, ongoing beneficial patient outcomes, and intervention adaptation to improve sustainment (both gray boxes) [5]. We theorize that hospitals’ *clinical capacity for sustainability*, which refers to the resources needed to sustain an intervention, is the primary set of determinants of intervention sustainability. To promote sustainability, interventions may be adapted or capacity may be changed to support ongoing intervention use, resulting in a feedback loop between the intervention and capacity over time.

The clinical context for this study includes both the hospital and pediatric oncology unit. To conceptualize the clinical context in sufficient detail, we use the Clinical Capacity for Sustainability Model [48]. This model suggests that clinical capacity for sustainability falls within 7 domains: (1) engaged staff and leadership—frontline and administrative staff who are supportive of the intervention; (2) engaged stakeholders—other individuals, such as patients or parents, who are supportive of the intervention; (3) organizational readiness—organizational internal support and the resources needed to effectively manage the intervention; (4) workflow integration—how well the intervention fits into work that is done or will be done; (5) implementation and training—the process of implementing and training to deliver and maintain an intervention; (6) monitoring and evaluation—a process to evaluate the intervention to determine its effectiveness; and (7) outcomes and effectiveness—using monitoring and evaluation to determine outcomes for clinicians or patients.

In our conceptual model, clinical capacity for sustainability is the primary predictor of PEWS sustainability, including both PEWS sustainment and continued benefit to patient outcomes. Capacity growth, PEWS adaptation, and PEWS implementation will co-occur during the initial implementation process with support from Proyecto EVAT. Hospitals are encouraged to adapt some elements of PEWS to suit local capacity but are expected to maintain fidelity to the PEWS tool and how it is used in patient care. Once implementation is complete, hospitals sustain PEWS, including both PEWS use and fidelity, independently of Proyecto EVAT. Hospitals may continue to build capacity or experience capacity declines. We hypothesize that a hospital’s baseline capacity and ability to increase or maintain capacity (Aim 1) leads to a greater likelihood of PEWS sustainability (Aim 2). We expect PEWS to be sustained indefinitely, specifically by continuing to use and maintain fidelity to the PEWS tool, despite minor fluctuations in overall capacity, its individual components, or appropriate PEWS adaptation. Through PEWS sustainability, we expect maintenance of lower CDE mortality rates long-term (Aim 2). If capacity drops or organizations cannot use PEWS with fidelity, PEWS may not be sustained (i.e., abandoned), resulting in increased CDE mortality rates. By identifying challenges to capacity, we will develop sustainability strategies targeting these challenges, which will subsequently improve PEWS sustainability (Aim 3).

### Setting

**Fig. 2:**
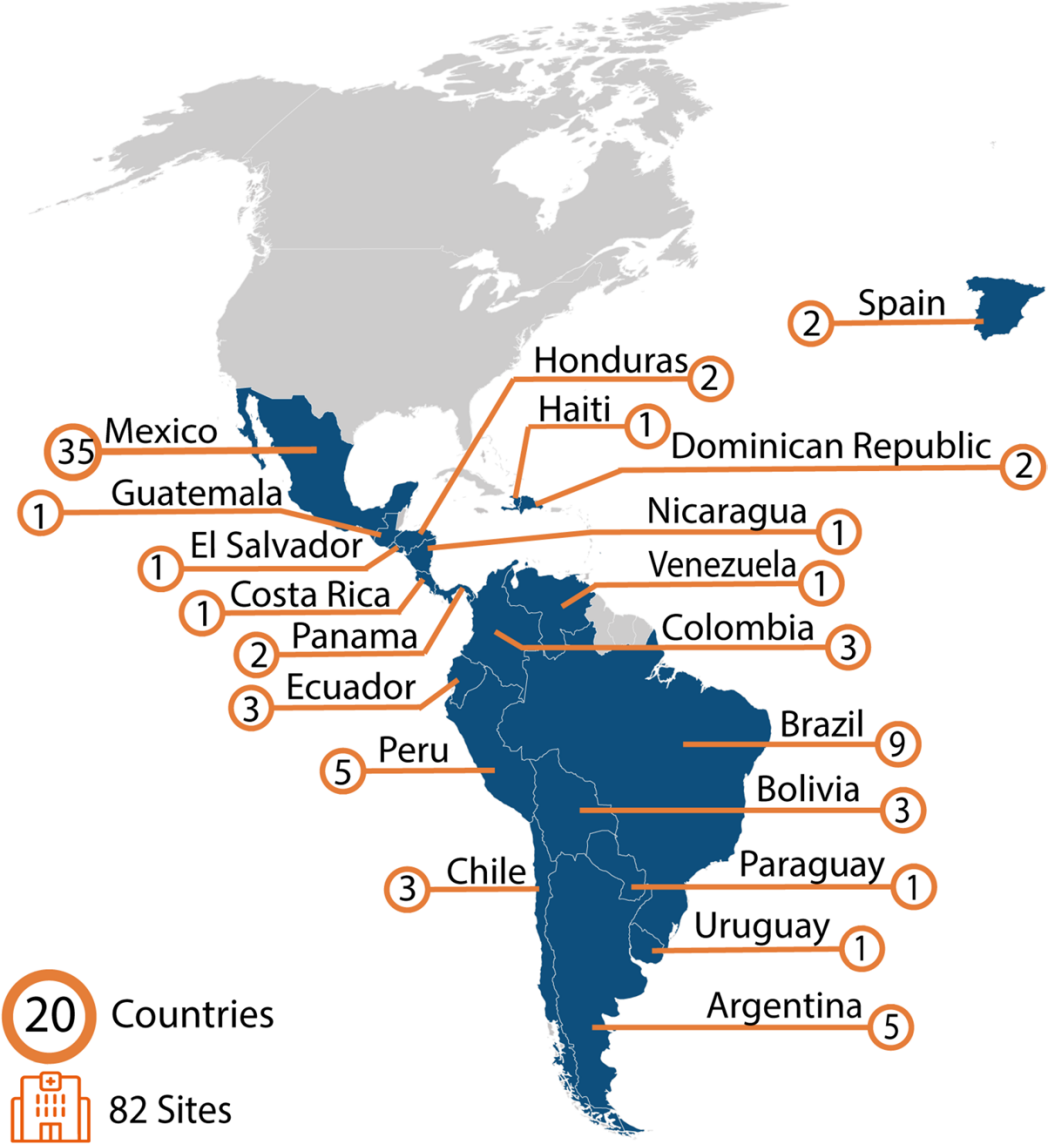
Proyecto EVAT: current hospitals and country locations

The proposed work will be conducted with resource-variable pediatric oncology hospitals participating in Proyecto EVAT. Currently, this includes 82 hospitals in 20 Spanish- and Portuguese-speaking countries in Latin America, representing over 10,500 new annual pediatric cancer diagnoses and more than 42,000 hospital admissions per year (Fig. 2). We expect that additional hospitals will be incorporated as we enroll new Proyecto EVAT cohorts during years 1 and 2 of the study period.

### Data collection

**Fig. 3:**
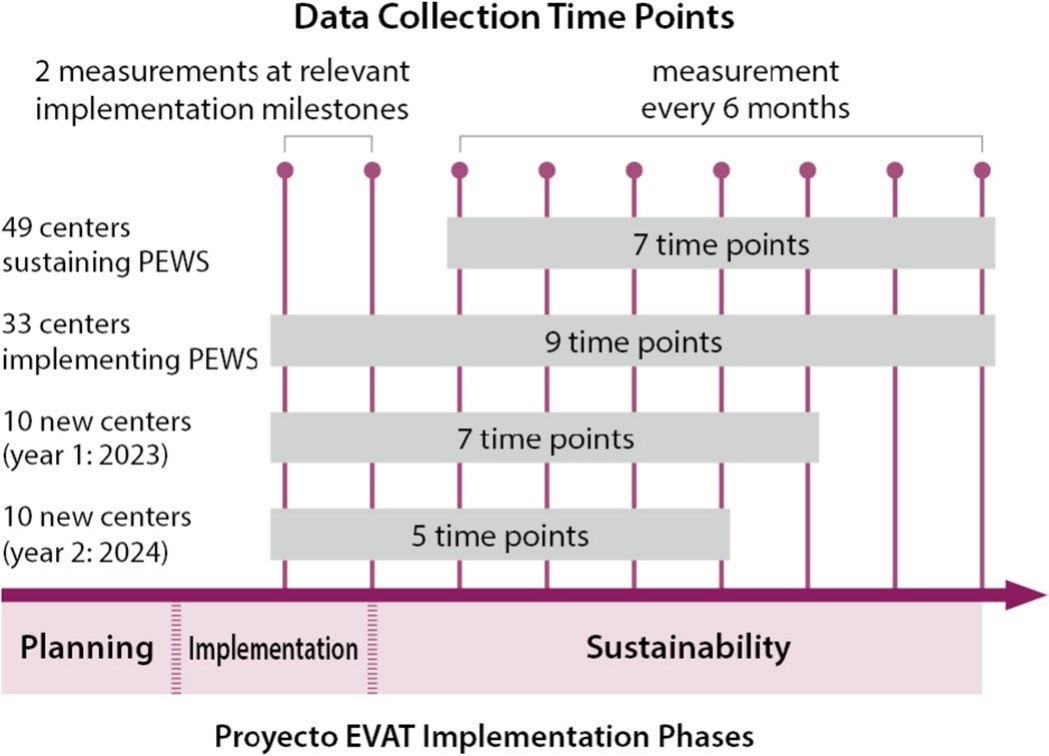
Study participation and data collection

For Aims 1 and 2, we will use a longitudinal observational research design. This will allow us to follow the development of capacity and the impact of capacity on PEWS sustainability in a variety of natural contexts. Over the study period, we will capture hospitals at various points in the PEWS implementation and sustainment process, ranging from those newly adopting PEWS to ones sustaining PEWS for over 10 years. We anticipate having between 5 and 9 observations of clinical capacity per hospital, dependent upon where hospitals are in the PEWS implementation process. We have structured data collection to occur at two relevant milestones in Proyecto EVAT to capture potential capacity increase during the adoption, initial implementation, and sustainment of PEWS. Once hospitals complete PEWS implementation and are sustaining PEWS, we will collect data every 6 months over the 4-year study data collection period (Fig. 3). We selected this interval to allow us to capture major changes in capacity and sustainability while minimizing participant burden. Among hospitals that have completed PEWS implementation, the primary outcomes, PEWS sustainment and CDE mortality rate, will be assessed in the 2 months prior to capacity assessments. For Aim 3, we will use a sequential mixed-method design by nesting qualitative data collection among hospitals who exhibit high and low capacity and have been using PEWS for at least 2 years. We will use focus groups of implementation leaders, clinicians, and hospital administrators to understand staff perspectives of the influence of capacity on PEWS sustainability and identify strategies that may develop capacity and support sustainability. We will then use an intervention mapping approach to identify critical capacity components and develop novel sustainability strategies for low-resource hospitals.

### Recruitment

All current Proyecto EVAT hospitals will be recruited for participation at the start of the project, and we expect to recruit two additional annual cohorts during the study period, resulting in approximately 102 study hospitals (82 current hospitals + 10 new hospitals per year × 2 years in subsequent cohorts). Each hospital’s local PEWS implementation leadership team will be contacted for participation in the study and asked to identify a site lead. Site leads will be responsible for obtaining hospital approval for participation and guide data collection at their hospitals. If hospitals do not wish to participate, they will have the option to opt out of the study but remain in Proyecto EVAT.

All PEWS implementation leadership team members (mean 7, range 4–15) and frontline clinical staff who routinely use PEWS (mean 20, range 9–61) will be eligible and invited to participate at each survey data collection time point (anticipated 27 participants per time point). Based on preliminary data, we conservatively expect at least a 50% response rate (currently 65%), resulting in at least 13 responses per time point (current mean is 19 per hospital).

### Study measures

Study measures and collection methods are summarized in Table 2 which aligns with our conceptual model. In alignment with our concept of sustainability, our primary outcomes are PEWS sustainment (i.e., sustainment outcome) and CDE mortality rate (i.e., patient outcome). Our primary quantitative covariates and predictors include hospital characteristics, participant characteristics, clinical capacity for sustainability, and PEWS adaptation.

**Table 2:** Overview of study measures

Data domain	Measures	Methods
Hospital Characteristics	Hospital type; funding; bed capacity; pediatric oncology and ICU staffing; annual volume of new diagnoses; and dates of PEWS adoption, training, pilot, and implementation completion.	• Collected at entry into Proyecto EVAT, confirmed by local site lead at study enrollment.
Participant Demographics	6 demographic questions on profession, role in PEWS implementation, years working in profession, gender, age, and hospital of employment.	• Anonymous Spanish or Portuguese paper or electronic Qualtrics survey, requiring 10–15 min to complete. • Distributed to (estimated *n* = 27 per hospital): • PEWS implementation leadership team (nurses and physicians, mean *n* = 7, range 4–15). • Frontline clinical staff (all ward and ICU physicians and nurses using PEWS, mean *n* = 20, range 9–61). • Participants allowed 3 weeks to complete survey with 3 reminders.
Clinical Capacity for Sustainability	CSAT: 7 domains with 5 questions (35 total), ranked on a 5-point Likert scale (1 = low to 5 = high agreement). Domain scores are the mean of domain items, and overall score is the mean of all domain scores; higher scores represent greater capacity.	
PEWS Adaptation	3 close-ended and 1 open-ended question on adaptations made to the PEWS scoring tool and algorithm [49]. Reported adaptations will be summed per respondent then averaged across all participants at each hospital.	
PEWS Sustainment	PEWS use and fidelity indicated by 3 types of errors (omission, scoring, and algorithm) in the 2 months before each CSAT data collection following implementation completion (sustainability phase). • Errors: (1) *omissions* (documented vital signs without using PEWS), (2) errors in PEWS *scoring*, and (3) PEWS *algorithm* nonadherence. • Dichotomous (yes/no < 15% in all three error types) and continuous (% errors).	• Assess by the implementation team by reviewing nursing documentation of vital signs and PEWS in all hospitalized patients. • Assessed weekly during implementation until implementation completion. • Assessed for 2 months prior to survey assessment during the sustainability phase. • Submitted electronically. • Data aggregated to calculate monthly error %.
Patient Outcomes	• CDE: an unplanned transfer to a higher level of care (i.e., ICU), use of an ICU intervention on the ward (vasoactive infusion, invasive or noninvasive mechanical ventilation, or cardiopulmonary resuscitation), or a ward death in a patient without limitations on resuscitation [10]. • CDE mortality rate: percent of death occurring during event or within 24 h of event conclusion [10]. • Reported for 2 months before each CSAT data collection time point during sustainability phase.	• Prospective de-identified registry of all CDEs in hospitalized pediatric oncology patients collected by the PEWS implementation team from start of participation in Proyecto EVAT. • For each CDE, a de-identified case report form is completed by local site leads and entered into a RedCAP database [50]. • Data analysis checks for missing and incorrect values assure data quality.
Challenges to Capacity and Sustainability	Perspectives from hospital staff on challenges to capacity development, PEWS adaptation, and PEWS sustainability, as well as possible interventions to support sustainability.	• Focus groups of 5–7 physicians, nurses, and hospital administrators (3 focus groups per hospital) at 4 high- and 4 low-capacity hospitals as assessed by the CSAT.

We operationalize PEWS sustainment as 2 consecutive months of PEWS use and fidelity, defined as < 15% PEWS errors (Table 2). Based on our prior work, we expect that some hospitals will have errors above this threshold and thereby be considered not sustaining. We will follow these hospitals to understand whether they resume PEWS sustainment or experience continued decline and PEWS abandonment. The primary patient outcome will be measured by the CDE mortality rate, calculated based on the de-identified quality improvement CDE registry (Table 2). We selected this patient outcome because it is the most reliable, easily collected, and most reflective of the effects of PEWS on patient care [44]. Implementation leaders will collect sustainment and CDE mortality data for 2 months before each data collection time point.

This study leverages the Clinical Sustainability Assessment Tool, a reliable measure based on the Clinical Capacity for Sustainability Model, to evaluate clinical capacity for sustainability among the Proyecto EVAT hospitals. The team developed the CSAT to assess the sustainability of clinical practices across 7 domains specific to health care and clinical settings [51]. Initial testing of the CSAT showed excellent internal consistency and preliminary evidence for discriminant validity (i.e., differences in CSAT scores by academic vs. nonacademic organizations and by inpatient vs. outpatient settings) and took about 15 min to complete [48, 51]. A Spanish version of the CSAT measure and associated report has been translated, regionally adapted, and validated for use in low-resource settings. An electronic version of the tool was piloted among 19 EVAT SC members to establish acceptability within the context of Proyecto EVAT, and feedback was used to create the final tool [49]. This work confirms the CSAT is culturally and contextually appropriate and discriminates between high- and low-capacity hospitals. Based on positive participant feedback, the CSAT was integrated into the Proyecto EVAT timeline in 2021, with two standardized CSAT measurements during the PEWS implementation phase (1: after the PEWS pilot to inform full-scale PEWS implementation, and 2: at implementation completion).

### Aim 1 analyses: changes in clinical capacity to sustain PEWS over time

We will investigate how overall capacity and its components change through the phases of PEWS adoption, implementation, and sustainment. We hypothesize that capacity will develop during early implementation and increase over time using PEWS. Primary data for Aim 1 will be hospital characteristics, participant demographics, and clinical capacity. Data will be examined for missingness and outliers and tested for normality, linearity, and homoscedasticity. Corrective strategies will be used as appropriate but may not be necessary given the robustness of mixed-effects modeling to various assumption violations [50, 52]. Data will be analyzed to generate descriptive statistics (e.g., frequencies, central tendencies, and variabilities) and diagnostic plots (e.g., bar charts and contingency tables) of capacity. Descriptive analyses will include variability of capacity across hospitals over time. All data management and analyses will be conducted using R (v4.3.1 or later).

We will use a multi-level modeling approach to build a series of growth curve models of capacity (CSAT scores) over time. The growth curve models will allow us to assess individual- and hospital-level associations with changes in capacity. Moreover, because capacity will be measured at many time points [5–9], the growth curve models will allow us to identify the linear and nonlinear change patterns of sustainability [52]. This rich description of changes in capacity is an important contribution of this study. The multi-level model template that will be used for these growth curve analyses is: ${SustCap}_{tij}={{IC}_{tij}+{HC}_{tj}+ IC}_{tij}*{HC}_{tj}$, where *SustCap* is capacity measured by the total or domain-specific CSAT scores; *IC* is the set of individual-level covariates (e.g., staff role); and *HC* is the set of hospital-level covariates (e.g., size). Time_0_ defines the start of the data collection; observations will be collected at time *t* for each outcome variable and time-varying covariate. The interaction term between the individual and hospital-level covariates allows us to explore cross-level interactions between setting and clinical staff characteristics. Multi-level modeling has many advantages for this type of organizational-level observational study [53]. It will allow us to build models that can appropriately handle staff- and patient-level data clustering within hospitals, examine the effects of both individual and hospital characteristics on the dependent variables, and analyze patterns of change over time.

### Aim 2 analyses: determine clinical capacity components that predict PEWS sustainability

We will identify capacity components that influence long-term sustainability. We hypothesize that greater overall capacity makes PEWS sustainment and continued benefits to patient outcomes more likely. Key variables include hospital characteristics, clinical capacity, PEWS adaptation, PEWS sustainment, and patient outcomes (Table 2).

We will follow a similar modeling strategy as described in Aim 1. We will build a series of multi-level growth curve models to assess changes in PEWS sustainment and patient outcomes over time, as a function of individual- and hospital-level, as well as capacity characteristics. As above, multi-level modeling has many advantages for this type of longitudinal study [53]. The first set of models will focus on PEWS sustainment as the primary outcome:${SustOutcome}_{tj}={{IC}_{tij}+{HC}_{tj}+ IC}_{tij}*{HC}_{tj}+{CSAT}_{tij}$, where *SustOutcome* measured at time *t* for hospital *j* is one of the PEWS sustainment variables (Table 2); *IC* is the set of individual-level covariates (e.g., staff role); *HC* is the set of hospital-level covariates (e.g., size), and CSAT is the set of total and domain-specific CSAT scores. Sustainment outcomes are either binary or percentages, so generalized mixed-effects modeling will be used [53]. The second set of models will then look at patient outcomes: ${PatientOutcome}_{tj}={{IC}_{tij}+{HC}_{tj}+ IC}_{tij}*{HC}_{tj}+{CSAT}_{tij}+{SustOutcome}_{tj}$. The interpretation of this model is similar to the previous one, but adding the PEWS sustainment outcomes as additional covariates. This will allow us to assess the extent to which success in PEWS sustainment is associated with downstream clinical outcomes. We will use either general or generalized mixed-effects modeling depending on the dependent variable (e.g., a *Poisson* model will be used for CDE mortality rates).

### Power estimates (Aims 1 and 2)

We used a simulation approach for power analysis in mixed-effects models according to the proposed analytic models [54]. We selected 2 prototypic models for estimating power: a *multilevel* model in which individual clinical staff are nested within hospitals (corresponding to secondary research questions that include cross-sectional multilevel analyses) and a *longitudinal* model in which hospital-level covariates and outcomes are measured over time (corresponding to the primary research questions in Aims 1 and 2). We obtained parameter estimates from study design decisions (e.g., number of hospitals) and analysis of the pilot data (e.g., means and variability of CSAT scores and hospital CDE mortality rates). We used conservative estimates of the number of participants, hospitals, time points, and intraclass correlation values. For the longitudinal models, we will have between 5 and 9 observations for each hospital. For power analyses, we assume 7 time points, which is a conservative estimate of the minimum number of observations we will have from most hospitals.

We conducted the power analyses with the SIMR package in R (Table 3) [55]. For each prototypic model, we calculated the power for detecting small or medium effect sizes for level 1, level 2, and cross-level interaction effects (L1, L2, CLI, respectively). Small and medium effect sizes are based on standardized estimates [55, 56]. The estimated power for the study ranged from good to excellent. Although the L2 main effects had lower power due to the number of hospitals, the more important effects for both models were CLIs (e.g., how clinical outcomes vary over time for different types of hospitals), which were excellent (> 95%) for both analyses.

**Table 3:** Power analyses for proposed analytic models

Effect type	Effect size	Power	95% CI
*Multilevel (13 participants in 90 hospitals)*			
L1–Staff	Small	100	99–100
L2–Hospital	Medium	91	82–96
CLI–Staff by hospital	Small	100	97–100
*Longitudinal (7 time points in 90 hospitals)*			
L1–Time	Small	94	88–98
L2–Hospital	Medium	83	75–89
CLI–Time by hospital	Small	95	89–99

### Aim 3: develop strategies to target clinical capacity and sustainability challenges

We will evaluate the perspectives of clinical staff and hospital administrators on capacity development, PEWS sustainment, and impact on patient outcomes in a subset of Proyecto EVAT hospitals exhibiting high- and low-capacity for sustainability. Using a sequential mixed-methods design, we will *qualitatively* determine staff perspectives on changes to capacity over time and how this relates to PEWS sustainment and patient outcomes. We will triangulate this with our *quantitative* assessment of capacity to provide a deeper understanding of how capacity relates to sustainability. We will then use an established implementation mapping process [57] to develop novel strategies to support PEWS sustainability in low-resource hospitals and address identified capacity challenges.

#### Recruitment and enrollment

Three focus groups (physicians, nurses, and administrators, separately) will be conducted at each of 8 Proyecto EVAT hospitals that have been using PEWS for at least 2 years (24 focus groups). The hospitals will be sampled purposively with a modified positive and negative deviant approach [58] to include four high-capacity and four low-capacity hospitals (using upper and lower quartiles of CSAT scores to recruit two high- and low-capacity hospitals in years 2 and 3). Based on our prior work indicating variation in capacity, this approach will allow us to explore how differences in capacity relate to sustainability and staff perspectives on identified capacity challenges. We will recruit participants using a purposive sampling approach [59] to include implementation leaders and clinical staff recruited for Aim 1 and 2, and hospital administrators identified by site leads, aiming to enroll 5–7 participants per focus group (total 120–168 participants). We will use homogenous grouping by participant roles to help ensure honest discussions [60, 61].

#### Data collection

Focus groups will be conducted using the video conferencing platform Zoom in Spanish or Portuguese by two native-speaking facilitators from St. Jude unknown to participants, and audio-recorded [49, 62]. The facilitation guide will be based on our conceptual framework (Fig. 2) and assess perceived challenges to capacity in the 7 CSAT domains, PEWS adaptation, PEWS sustainment, impact on patient outcomes and potential strategies to promote sustainability. We expect focus groups to last 60–90 min.

#### Analysis plan

Audio recordings will be translated into English and transcribed through a certified service [31–33, 62, 63]. English transcripts will be de-identified, segmented, and uploaded to MAXQDA software for analysis. A qualitative analysis team will develop an initial codebook with a priori codes informed by the CSAT domains and conceptual framework as well as inductive codes developed using a constant comparative approach with iterative memoing of transcripts to allow for emergent themes [64]. Transcripts will be coded independently by two coders. Interrater reliability will be monitored, and discrepancies resolved through consensus and a separate adjudicator. We expect to use two broad analytic approaches: categorical coding, which will group data conceptually according to the domains of our framework, and thematic coding, which will describe the relations among the concepts (e.g., the dynamic between capacity and PEWS sustainability) [65]. Because our approach is guided by a structured framework and a previously employed method for inductive codes, we are confident that this strategy will achieve analytic saturation [66].

#### Data synthesis

The results from *quantitative* assessment of capacity using the CSAT will be triangulated with *qualitative* participant perspectives on capacity development and PEWS sustainability to provide convergence (i.e., to assess how different data answer the same question) [67, 68]. We will further explicate primary quantitative findings through joint displays, facilitating comparisons of quantitative and qualitative results [69]. Specifically, qualitative results will be used to gain a deeper understanding of capacity strengths and challenges, as well as how capacity relates to PEWS sustainability [70, 71].

#### Strategy development

In year 5, we will use results from the above analyses to develop sustainability strategies by leveraging implementation mapping, which applies intervention mapping to implementation strategy development [57]. To date, sustainability strategies for clinical settings have been primarily developed based on literature review and without a systematic process [11]. Intervention mapping is widely used to design and adapt behavioral interventions and provides a systematic process to development interventions using five steps: (1) conduct a needs assessment; (2) identify sustainability outcomes, performance objectives, determinants, and create matrices of change; (3) choose theories of change and select strategies; 4) produce strategy protocols and materials; (5) evaluate outcomes [72, 73]. Our activities and analyses from the three study aims will serve as the needs assessment and address step 1: identify capacity barriers and needs. To accomplish the second step, our research team will use study results to identify performance objectives and create matrices of change. Performance objectives are actions that will accomplish the intended sustainability outcome. For instance, a simple performance objective to improve PEWS sustainment would be to have nurses use PEWS more often. Matrices of change are an analytic technique that help integrate determinants, theories of change, performance objectives, and sustainability outcomes to select strategies that directly address critical determinants in a theoretically-sound manner (third step). For instance, we may identify nurse turnover and lack of PEWS knowledge (determinants) as primary barriers to PEWS use among nurses (performance objective) impacting overall PEWS sustainment (outcome). To address this barrier, we may select an educational strategy, based on the theoretical assumption that knowledge leads to behavior change, such as booster PEWS training sessions for new nurses. We will present our strategy development progress to the EVAT SC for feedback. By the end of the study period, we will have developed materials and protocols needed for these strategies (fourth step) using recommendations for specifying strategies [74] and be positioned to evaluate these strategies (step 5) in future work using a design, such as a hybrid type III trial, appropriate for strategy evaluation.

### Dissemination plan

Our dissemination plan considers various audience, including researchers, hospital administrators, clinical staff, and funders. We will share our findings through conference presentations and open-access publications. All versions of the CSAT are publicly available at the *SustainTool.org* website [75], which will be updated with study results and resources for study partners. Individual center results of the capacity assessment will be shared in real-time with centers using the CSAT reports [49]; we will also develop an interactive dashboard to share findings and developed strategy materials with study participants and the public. Making these tools freely available will expedite their use as common measures in future studies and by clinicians and other stakeholders looking to assess the clinical capacity of their organizations.

## Discussion

The INSPIRE study prospectively follows capacity and its impact on sustainability over time. By choosing this design, we accept loss of control over recruitment and measurement conditions. However, our design is strengthened by the diversity in location, size, and capacity of the participating hospitals, allowing us to longitudinally follow the natural course of PEWS sustainability over many years. A longitudinal observational study is the most appropriate design to provide empirical evidence for the interrelations between capacity, PEWS sustainment, and patient outcomes in *real-world* low-resource hospitals.

Sustainability of evidence-based interventions is perhaps the *most important aspect* of the implementation continuum, yet has not been rigorously examined, particularly in low-resource settings [10]. This study will address this significant scientific gap by moving beyond conceptual frameworks to empirical testing to understand the relationship between clinical capacity, intervention sustainment, and patient outcomes in variably-resourced hospitals over time. A better understanding of how to sustain evidence-based interventions like PEWS is urgently needed to increase global survival of childhood cancer, particularly in low-resource settings. Upon completing the proposed work, we will establish how capacity changes over time, determine its impact on intervention sustainment and patient outcomes, and use staff perspectives on capacity building to develop novel sustainability strategies. This work is significant by providing a theoretically driven, longitudinal understanding of factors that predict sustainability in a large cohort of low-resource hospitals delivering the same intervention in a variety of settings. It is innovative by moving beyond a cross-sectional exploration towards empirical, longitudinal evidence supporting the dynamic relationships between capacity and intervention sustainability. Furthermore, we will leverage this knowledge to address a widely identified need to develop sustainability strategies that optimize capacity, promote intervention sustainability, and encourage health equity in childhood cancer outcomes in low-resource settings [1, 76]. Ultimately, these results will launch a trajectory of research that improves childhood cancer survival by effectively sustaining evidence-based interventions like PEWS and promoting equity by focusing on low-resource hospitals where preventable mortality remains high.

## Data Availability

Not applicable.

## Abbreviations

CDEs: Clinical deterioration events
CLI: Cross level interaction
CSAT: Clinical Sustainability Assessment Tool
EB: Evidence-based
EVAT: Escala de Valoración de Alerta Temprana
EVAT SC: EVAT Steering Committee
INSPIRE: *IN*Vestigating *S*ustainability of *P*EWS In *RE*source-limited hospitals
L1: Level one
L2: Level two
PEWS: Pediatric Early Warning System
PICU: Pediatric intensive care unit
WHO: World Health Organization
